# Measuring the global burden of chikungunya and Zika viruses: A systematic review

**DOI:** 10.1371/journal.pntd.0009055

**Published:** 2021-03-04

**Authors:** Christopher J. Puntasecca, Charles H. King, Angelle Desiree LaBeaud

**Affiliations:** 1 Stanford University School of Medicine, Stanford, California, United States of America; 2 Center for Global Health and Diseases, Case Western Reserve University School of Medicine, Cleveland, Ohio, United States of America; National Research Centre, EGYPT

## Abstract

Throughout the last decade, chikungunya virus (CHIKV) and Zika virus (ZIKV) infections have spread globally, causing a spectrum of disease that ranges from self-limited febrile illness to permanent severe disability, congenital anomalies, and early death. Nevertheless, estimates of their aggregate health impact are absent from the literature and are currently omitted from the Global Burden of Disease (GBD) reports. We systematically reviewed published literature and surveillance records to evaluate the global burden caused by CHIKV and ZIKV between 2010 and 2019, to calculate estimates of their disability-adjusted life year (DALY) impact. Extracted data on acute, chronic, and perinatal outcomes were used to create annualized DALY estimates, following techniques outlined in the GBD framework. This study is registered with PROSPERO (CRD42020192502). Of 7,877 studies identified, 916 were screened in detail, and 21 were selected for inclusion. Available data indicate that CHIKV and ZIKV caused the average yearly loss of over 106,000 and 44,000 DALYs, respectively, between 2010 and 2019. Both viruses caused substantially more burden in the Americas than in any other World Health Organization (WHO) region. This unequal distribution is likely due to a combination of limited active surveillance reporting in other regions and the lack of immunity that left the previously unexposed populations of the Americas susceptible to severe outbreaks during the last decade. Long-term rheumatic sequelae provided the largest DALY component for CHIKV, whereas congenital Zika syndrome (CZS) contributed most significantly for ZIKV. Acute symptoms and early mortality accounted for relatively less of the overall burden. Suboptimal reporting and inconsistent diagnostics limit precision when determining arbovirus incidence and frequency of complications. Despite these limitations, it is clear from our assessment that CHIKV and ZIKV represent a significant cause of morbidity that is not included in current disease burden reports. These results suggest that transmission-blocking strategies, including vector control and vaccine development, remain crucial priorities in reducing global disease burden through prevention of potentially devastating arboviral outbreaks.

## Author summary

Chikungunya and Zika are 2 mosquito-borne viral diseases that can cause both acute symptoms and long-term, debilitating complications in infected individuals. Chikungunya is best known as a cause of persistent arthritis in otherwise recovered patients and Zika as a cause of cognitive, motor, and sensory anomalies in newborn children. Both diseases emerged in the Americas within the last decade and have since spread rapidly throughout the region. Despite their widespread transmission there and throughout much of the world, chikungunya and Zika remain neglected diseases. One of the most significant obstacles to address their spread is a lack of data involving their burden. We searched the published literature and surveillance reports to collect information about the incidence, mortality, and morbidity associated with each of these diseases to estimate their regional and global burden during the last decade. Our estimates confirm that chikungunya and Zika caused substantial burden throughout this time frame and place them among the most problematic mosquito-borne viral diseases worldwide. We found that the largest proportion of global burden linked to each disease between 2010 and 2019 occurred in the Americas, although this observation is likely due to limited reporting in other regions.

## Introduction

Arthropod-borne viruses, or arboviruses, are exceedingly common causes of disabling fever syndromes worldwide, but the cumulative burden of disease that they cause is not well quantified [1]. Acutely, arboviral infections can range from asymptomatic to debilitating and undifferentiated febrile illnesses. They can also progress to severe secondary conditions or cause congenital maladies, both of which may result in long-term physical disability, cognitive impairment, or early death [2–4].

Among over 100 arboviruses that cause disease in humans [2], chikungunya virus (CHIKV) and Zika virus (ZIKV) have been particularly problematic in the last decade due to their rapid emergence in the Western Hemisphere [5,6]. CHIKV, an alphavirus of the Togaviridae family, caused several outbreaks throughout the Pacific region in the early 2000s. The virus then spread to the Americas in 2013, when the first reported autochthonous case of CHIKV was reported in Brazil [7]. CHIKV has since infected millions across all 6 World Health Organization (WHO)-defined regions [8–10].

ZIKV, a flavivirus of the family Flaviviridae, has been known to cause disease in humans since the 1950s [11,12]. The spread of ZIKV, however, remained minimal until 2007, when the first major Zika outbreak occurred in the Federated States of Micronesia [11,12]. ZIKV continued to cause outbreaks in other Pacific and Southeast Asian nations between 2012 and 2014. Similarly to CHIKV, the virus’s spread through the Americas began in Brazil, where the first autochthonous ZIKV infection was reported in 2015. Hundreds of thousands have been infected with the virus since its emergence in the Western Hemisphere, and a number of regions across the globe remain at high risk for future outbreaks [5,10,13].

As with other arboviruses, the disabling sequelae of CHIKV and ZIKV disproportionately affect resource-poor communities, where they frequently cause chronic impairment that can greatly reduce patients’ qualities of life [2,14,15]. Its name being derived from a Makonde word meaning “that which bends up,” chikungunya has been characterized since the 1950s as a cause of persistent polyarthralgias among those infected [16]. Recent studies have revealed other lesser known clinical manifestations of CHIKV that can contribute to otherwise unrecognized burden, including medium- to long-term neurologic symptoms and inflammatory ophthalmic complications like uveitis [17,18]. Intrapartum CHIKV transmission has also been observed and can cause neonatal encephalitis and poor neurodevelopmental outcomes [19–21].

While the acute phase symptoms of ZIKV infection are often less severe than those of chikungunya, the virus can cause severe, often life-altering complications in some patients [22]. Several studies have elucidated a link between ZIKV infection and Guillain–Barré syndrome (GBS), although estimates of progression to GBS vary between reports [23–26]. The most concerning feature of ZIKV, however, is its teratogenic effect on fetal neurologic development, which manifests as a complex syndrome characterized by microcephaly at birth, severe motor and cognitive impairment, frequent seizures, ocular defects, and auditory deficits [27–30]. Since congenital Zika syndrome (CZS) has only been a recognized complication since 2015, longer-term prospective follow-up will be necessary to further describe the condition and study the progression of symptoms throughout life [31]. While long-term prognosis remains unknown, data examining large CZS cohorts confirm that the syndrome causes considerable burden for both affected children and their caretakers at least through 3 years of age [28,32,33]. As of 2020, a WHO project examining pregnancy and child outcomes among ZIKV cohorts from many regions is currently ongoing [34].

The most recent Global Burden of Disease (GBD) estimates published by WHO and the Institute for Health Metrics and Evaluation (IHME) include assessments of only 3 arboviral diseases: dengue, yellow fever, and Zika [35]. For other arboviruses, including CHIKV, all cause-specific mortality and long-term infection-related morbidity represent substantial health deficits omitted in international disease burden reports and are consequently not included in top-level discussions of disease control priorities [36]. Although estimates for 2019 ZIKV-related burden are included in the most recent GBD report, data from other years remain absent. With evidence from the past decade indicating the high incidence and frequency of disabling sequelae due to CHIKV and ZIKV, it is important to address this gap in knowledge and to quantify the impacts of the 2 viruses. In the present study, we reviewed the available data detailing CHIKV and ZIKV infections and their impact in order to estimate the disability-adjusted life years (DALYs) lost globally as a result of their spread over the last decade.

## Methods

### Search strategy

We systematically reviewed the available published literature and official reports on CHIKV and ZIKV. Searching was initiated with the use of the arbovirus name and the terms “outbreak(s),” “complication(s),” “disability,” “quality of life,” “morbidity,” “mortality,” “DALY,” and “QALY” in PubMed, Google Scholar, LILACS, African Journals Online, SciELO, and Web of Science. The specific search algorithms used in each database are included in S1 Text. Bibliographies of selected publications were also searched for additional reports. English, Spanish, French, and Portuguese reports were screened. A complete listing of included primary research articles can be found in S1 Table. Additional epidemiologic surveillance data from national, regional, and global reporting bodies were referenced to generate case count estimates. These reports include those published by both the United States and European Centers for Disease Control and Prevention, WHO, Pan American Health Organization (PAHO), ProMed, and national ministries of health and detail the incidence of CHIKV and ZIKV throughout their respective regions between 2010 and 2019. A complete listing of included surveillance reports can be found in S2 Table.

### Study selection

Publications were required to meet 3 inclusion criteria: (1) discussion of complications that lead to mortality or prolonged morbidity; (2) focus on population-based information; and (3) reported data collected between 2010 and 2019. In order to assess the population-level impacts of endemic disease, studies involving travelers from non-endemic areas and all case reports were excluded from the analysis. Reviews and other sources that reported secondary data were excluded from the analysis. Studies and surveillance reports that satisfied preliminary screening were evaluated to ensure that data were not duplicated in other included studies.

### Data analysis

In order to derive DALY estimates for CHIKV and ZIKV, we extracted from the included reports estimates of incidence, mortality, average age at death, and, for nonlethal cases, information on the duration and severity of acute and chronic symptoms. Study populations and dates were carefully reviewed to ensure that any duplicate data were not included more than once.

DALY estimates for each disease were calculated according to standard methods outlined in the GBD guidelines [37] using spreadsheets developed by the authors. These spreadsheets, included as S3–S6 Tables, are programmed to generate DALY estimates using the formulae listed below. DALY scores for each condition represent the sum of 2 components: (a) for cases of mortality, the years of healthy life lost (YLL) from a standard expected years of life lost (SEYLL), plus (b) for individuals having nonlethal, disease-specific disability, the years lived with disability (YLD) multiplied by a disability weight (DW) reflecting the proportion of impairment caused by that health condition [37]. Because of limitations in the available data, our summary estimates were calculated for both sexes together and not distributed according to age group, as ideally presented in DALY tables [35,37]. We report our DALY estimates in 2 formats: discrete approximations calculated using inputs derived from weighted averaging of the variables extracted from included publications and ranges based on the variability of credible input values contained in those reports.

DWs for acute ZIKV disease, subsequent GBS, and CZS were taken from James and colleagues (Table 1) [38]. For acute CHIKV infection, the range of DWs was based on published values for the analogous febrile syndromes caused by dengue and yellow fever [38]. The range of DW for chronic sequelae following CHIKV infection was the published DWs for mild to severe rheumatoid arthritis, conditions analogous to persistent post-chikungunya rheumatic symptoms. In order to generate comprehensive burden assessments, the pervasive psychological sequelae reported among mothers and caretakers of children with CZS were also included in the analysis [33,34]. To calculate this burden, DWs for depression and anxiety were taken from James and colleagues [38], while the DW for post-traumatic stress disorder (PTSD), not listed in the GBD framework, was taken from Lim and colleagues [39]. To calculate YLD, DWs were multiplied by the relevant duration of symptoms, as reported in the literature for each condition.

**Table 1:** Summarized symptom frequency and related DWs.

Arbovirus	Disease details and estimated prevalence of complications	DW for condition(s) or analogous condition(s) from GBD project listings [38,39]	Average (and range) of DW values used for YLD estimations
CHIKV	Acute undifferentiated febrile illness which can include joint pain and rash [2,4]	Mild dengue or yellow fever, DW = 0.051; severe dengue or yellow fever, DW = 0.133	**0.092** (0.051–0.133)
	42.5% (7.0% to 89.7%) of survivors develop persistent postinfectious neurologic and rheumatic symptoms, including polyarthritis [50–56]	Mild rheumatoid arthritis, DW = 0.117; severe rheumatoid arthritis, DW = 0.581	**0.349** (0.117–0.581)
ZIKV	Acute undifferentiated febrile illness which can include joint pain, rash, and conjunctivitis [3,5,6]	Acute Zika infection, DW = 0.051	**0.051**
	0.020% (0.016% to 0.024%) of survivors develop postinfectious GBS, which can cause temporary or permanent paralysis and incontinence [22–24]	GBS due to Zika infection, DW = 0.296	**0.296**
	7.0% (3.4% to 14.4%) of children born to mothers infected with ZIKV during pregnancy will develop CZS, with likely lifelong cognitive, motor, visual, and hearing impairment with severe epilepsy [27–31,57–61]	CZS: combined DW (severe motor plus cognitive impairment, DW = 0.542 and severe epilepsy, DW = 0.552)	**0.795**
	High rates of psychological sequelae are associated with caring for surviving children with CZS: Among caretakers, 30% report moderate and 21% severe depression, and 20% report moderate and 31% severe anxiety; additionally, 17%, 34%, and 43% of mothers who experience EPL or stillbirth report depression, anxiety, and PTSD, respectively, often lasting several months [32,33,62,63]	Moderate or severe depression, DW = 0.396 or DW = 0.658	**0.527** (0.396–0.658)
		Moderate or severe anxiety, DW = 0.133 or DW = 0.523	**0.328** (0.133–0.523)
		PTSD, DW = 0.435	**0.435**

CHIKV, chikungunya virus; CZS, congenital Zika syndrome; DW, disability weight; EPL, early pregnancy loss; GBD, Global Burden of Diseases; GBS, Guillain–Barré syndrome; PTSD, post-traumatic stress disorder; YLD, years lived with disability; ZIKV, Zika virus.

To estimate the incidence of clinical CHIKV and ZIKV infections in affected countries, we used the cumulative case counts reported in available surveillance records and published reports (see S2 Table for complete listing of surveillance documents referenced). Lab-confirmed infections with the arboviruses served as the lower bounds for our case count estimates. These infections, combined with those documented as “suspected” or “likely” by the respective reporting bodies, served as the upper bound. In light of the recognized trends of underreporting [40–42], these larger case count values were used as the inputs for our calculations of discrete estimates. Approximate incidence rates were annualized by dividing cumulative cases from 2010 to 2019 by 10.

Per the GBD framework guidelines, final DALYs were calculated as follows:

DALY = YLL + YLD_acute_ + YLD_chronic_, where
YLL = (Incident deaths) × (standard expected years of life lost at median age of death)
YLD_acute_ = (Incident cases with acute disease only) × DW_acute_ × (duration acute disease)
YLD_chronic_ = (Incident cases progressing to chronic disease) × DW_chronic_ × (duration of chronic disease).

The combined DW for CZS was obtained using the multiplicative equation defined in the revised GBD framework from 2010, which recognizes the increased disability associated with comorbid conditions while precluding disabilities in excess of 1:

$$Combined\;DW=1–\left(1–DW_{a}\right)\;x\;\left(1–DW_{b}\right),\;where$$

DW_a_ and DW_b_ are the DWs for 2 distinct conditions occurring simultaneously.

As of 2010, the GBD framework calls for the use of 92 years as the standard for life expectancy, a value based on the frontier national life expectancy for 2050 among Japanese and Korean women [37].

## Results

**Fig 1:**
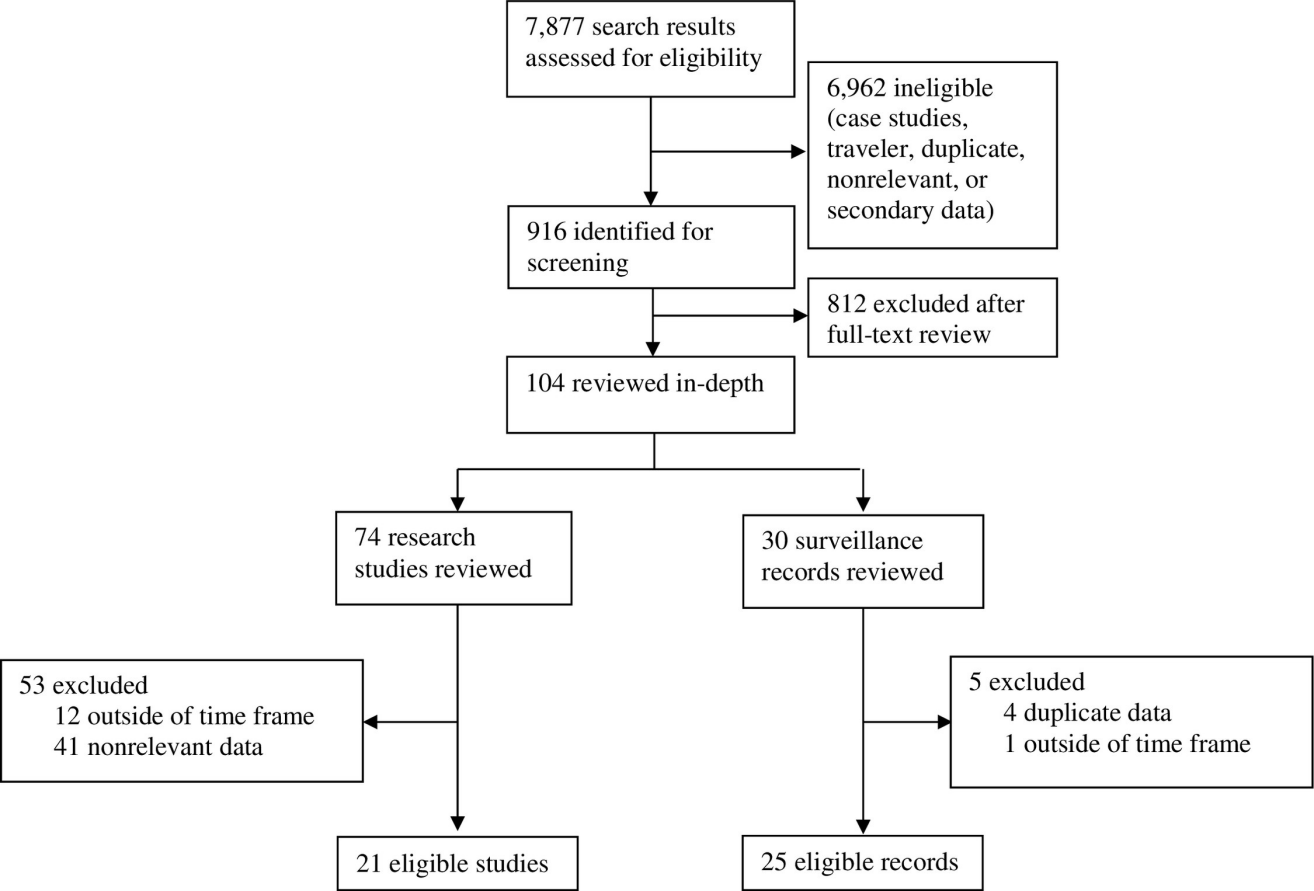
Study selection. Breakdown of initial search results returned, reports screened, and sources included in the analysis.

Out of 7,877 studies identified, 916 were screened, and 21 were selected for inclusion (Fig 1).

Tables 1 and 2 summarize the findings of the studies included in our analysis. CHIKV has historically been associated with relatively low mortality, and recent data gathered in the Americas have indicated that the case fatality rate (CFR) is likely slightly lower than 1 per 1,000 infections [42,43]. Surveillance data released by WHO in 2019, however, point to a significantly higher CFR among the Sudanese population, setting the value of 18 per 1,000 clinical infections as the credible upper bound for CFR [44]. Although the fever, rash, and joint pain associated with acute infection are usually self-limited and last only 1 to 2 weeks [45], CHIKV can result in neurologic [17], renal [46], cardiac [47], respiratory [48], and hematologic [49] complications that may result in death or necessitate aggressive medical interventions. Moreover, the acute symptoms of CHIKV often progress to long-term rheumatism, including severe arthritis that can persist for several years. A large proportion of patients who progress to these chronic symptoms seek medical treatment during the course of their disease, with variable success in symptom management [50].

**Table 2:** Extracted data used for DALY calculations.

Arbovirus	Estimated global clinical cases per annum*	CFR (%)	Median age for fatal cases	Survivor’s risk for chronic or permanent disability	Median age for symptomatic disease	Duration of chronic disease (years)
CHIKV	52,774 to 328,943	0.07% (0.012% to 1.8%) [40,42,44]	60 [50]	42.5% (7.0% to 89.7%) [50–56]	39 [50–55]	2.0 (1.0 to 53) [50–56]
ZIKV	25,344 to 99,365	0.0023% (0.0020% to 0.0026%) [64,65]	72 [56]	(0.020%) (0.016% to 0.024%) [23–25]	40.5 [23–25]	1.0 (0.083 to 52) [22–24]
ZIKV (CZS)	372 to 795	29.9% (27.3% to 64.4%) [27,61]	0 [28,61]	100%^†^	0 [22–26,40–42]	92 (1.7 to 92) [28,30,31,66,67]

CFR, case fatality rate; CHIKV, chikungunya virus; CZS, congenital Zika syndrome; DALY, disability-adjusted life year; ZIKV, Zika virus.

*See S2 Table for complete listing of surveillance reports used to generate case count estimates.

^†^Reported CZS cases are those that meet predefined international diagnostic criteria based on clinical symptoms. Current evidence suggests that these symptoms will persist throughout life; therefore, all of the CZS patients included in this analysis are assumed to have some degree of chronic or permanent disability [28,58,62].

Estimates of the rate of progression to chronic post-chikungunya rheumatism vary widely based on criteria used and the populations examined, with data indicating that, on average, about 42.5% of patients experience chronic, post-chikungunya arthritis [50–56]. Such symptoms can substantially diminish a patient’s quality of life, and formal studies have confirmed that post-chikungunya arthritis can severely impair activities of daily living [14,53].

The symptoms of acute ZIKV infection are similar to, but usually milder than those of CHIKV [22,38]. As with CHIKV, most patients acutely infected with ZIKV recover within 1 to 2 weeks [57]. The CFR associated with acute ZIKV infection in adults is also extremely low, with the majority of deaths occurring in older patients with preexisting comorbidities [64,65]. Nevertheless, acute ZIKV infection has been found to cause cardiovascular and other complications, with some otherwise healthy individuals requiring hospitalization and respiratory support during infection [68,69]. ZIKV has also been linked to GBS, a rare, paralyzing neurologic condition that can leave up to 45% of patients unable to walk without assistance 6 months after symptom onset. Studies indicate that the incidence of post-ZIKV GBS is about 2 per 10,000 ZIKV infections, making the arbovirus a leading cause of the condition among affected populations during outbreaks [23–26]. In addition to the acute febrile symptoms, mortality, and GBS associated with adult infections, ZIKV can also have severe teratogenic effects on fetal development, causing CZS.

CZS is associated with a complex constellation of symptoms that cause substantial morbidity and mortality among affected populations. Due to the ZIKV’s tropism for fetal neural progenitor cells, as many as 1 in 7 neonates whose mothers were infected with ZIKV during pregnancy develop neurologic, optic, or aural malformations [27–31]. CZS is classically characterized by microcephaly at birth, and this finding is a requirement for diagnosis in most settings [66,67]. However, the neurologic complications of CZS have also been documented in some normocephalic children, indicating that current surveillance may omit many cases of CZS [70]. Although research is limited, studies have demonstrated that the motor and cognitive impairments experienced by many children with CZS persist at least throughout infancy, with recent studies documenting substantial developmental delays at 3 years of age [28]. Longer-term follow-up is needed to characterize the progression of CZS symptoms beyond this age, although the severity of the related neurologic malformations make it likely that patients with CZS will experience lifelong symptoms [28,30,31,66,67]. Between 27.3% and 64.4% of CZS cases result in early pregnancy loss (EPL) or perinatal death [27,61]. While CZS-related morbidity most directly affects patients with the condition, a large amount of burden also arises from the disease’s impact on mothers and caretakers. The psychological sequelae experienced by the caretakers of children with CZS are extremely pervasive, with about half of surveyed caretakers, usually mothers, reporting moderate or severe depression and a similar proportion reporting anxiety [32,33]. Studies have also shown that over a third of mothers who experience EPL report depression, anxiety, or PTSD for months following the loss [62]. These psychological symptoms can all negatively impact quality of life and one’s ability to perform activities of daily living and so were consequently included in our ZIKV-related DALY estimates.

Using the values listed in Tables 1 and 2, we estimate that from 2010 to 2019, CHIKV caused an average annual global loss of over 106,000 DALYs, while ZIKV caused the average annual loss of over 44,000 DALYs. Both arboviruses caused significantly more burden in WHO’s Region of the Americas (AMRO) than in any other region, reflecting their rapid expansion there. We estimate that CHIKV and ZIKV caused the respective annualized loss of 94,995 and 42,690 DALYs in the Americas when averaged over the 10 years studied. However, these estimates increase substantially when only the years since each virus’s emergence in AMRO are considered. When only data from 2014 through 2019 are analyzed, CHIKV caused an annual average burden of over 158,000 DALYs in AMRO. Likewise, if only the 5 years since ZIKV’s emergence in the Americas are considered, the virus’s associated burden in the region effectively doubles to an annualized average of over 85,000 DALYs.

Tables 3 and 4 summarize the DALY estimates for CHIKV and ZIKV, detailing the relative contributions of acute disease, chronic complications, and early mortality. YLD due to chronic complications contribute substantially more to the overall DALY burden of CHIKV than the symptoms and mortality associated with acute infection. CZS cases, although representing a small proportion of the total number of Zika cases, account for the majority of ZIKV-related burden.

**Table 3:** DALY estimates for CHIKV.

	YLL_acute_	YLD_acute_	YLD_chronic_	TOTAL*
AFRO	64 (0–2,548)	10 (0–14)	814 (1–74,360)	**888** (1–75,637)
AMRO	6,592 (312–275,704)	1,089 (5–1,457)	87,314 (401–7,977,910)	**94,995** (767–8,111,092)
EMRO	32 (0–1,820)	7 (0–10)	580 (16–52,960)	**619** (18–53,833)
EURO	0 (0–52)	0 (0–2)	9 (0–840)	**9** (0–865)
SEARO	544 (0–22,308)	88 (0–118)	7,066 (7–645,676)	**7,699** (8–656,457)
WPRO	128 (0–5,460)	22 (0–29)	1,729 (0–158,012)	**1,879** (0–160,658)
TOTAL*	7,360 (312–307,892)	1,216 (5–1,627)	97,513 (424–8,909,812)	106,089 (794–9,058,541)

AFRO, WHO Africa region; AMRO, WHO Americas region; CHIKV, chikungunya virus; DALY, disability-adjusted life year; EMRO, WHO Eastern Mediterranean region; EURO, WHO European region; SEARO, WHO Southeast Asia region; WPRO, Western Pacific region; YLD, years lost to disability; YLL, years of life lost.

*Note: Total DALY min. and max. values do not sum across rows because gains in 1 DALY category column are necessarily offset by reductions in other categories.

**Table 4:** DALY estimates for ZIKV.

	YLL_acute_	YLD_acute_	YLL_GBS_	YLD_GBS_	YLL_CZS_	YLD_CZS_	YLD_care_	YLD_EPL_	TOTAL
AFRO	-	1 (1–2)	-	-	92 (0–276)	146 (0–219)	48 (0–90)	-	**287** (1–403)
AMRO	40 (40–60)	96 (23–197)	108 (0–206)	5 (0–289)	12,236 (9,384–45,632)	22,734 (178–40,958)	7,460 (118–17,364)	16 (4–59)	**42,690** (10,058–77,884)
EMRO	-	-	-	-	-	-	-	-	-
EURO	-	-	-	-	0 (0–92)	73 (0–73)	24 (0–30)	-	**97** (0–104)
SEARO	-	-	-	-	0 (0–92)	73 (0–73)	24 (0–30)	-	**97** (0–173)
WPRO	-	2 (2–5)	-	0 (0–15)	276 (0–1,104)	512 (0–951)	168 (0–391)	0 (0–1)	**958** (2–1,812)
TOTAL	40 (40–60)	100 (26–203)	108 (0–206)	5 (0–304)	12,604 (9,384–47,104)	34,772 (178–42,275)	7,724 (118–17,364)	16 (4–61)	44,130 (10,061–80,407)

AFRO, WHO Africa region; AMRO, WHO Americas region; care, caregiver impact; CZS, congenital Zika syndrome; DALY, disability-adjusted life year; EMRO, WHO Eastern Mediterranean region; EPL, early pregnancy loss; EURO, WHO European region; GBS, Guillain–Barré syndrome; SEARO, WHO Southeast Asia region; WPRO, Western Pacific region; YLD, years lost to disability; YLL, years of life lost; ZIKV, Zika virus.

**Fig 2:**
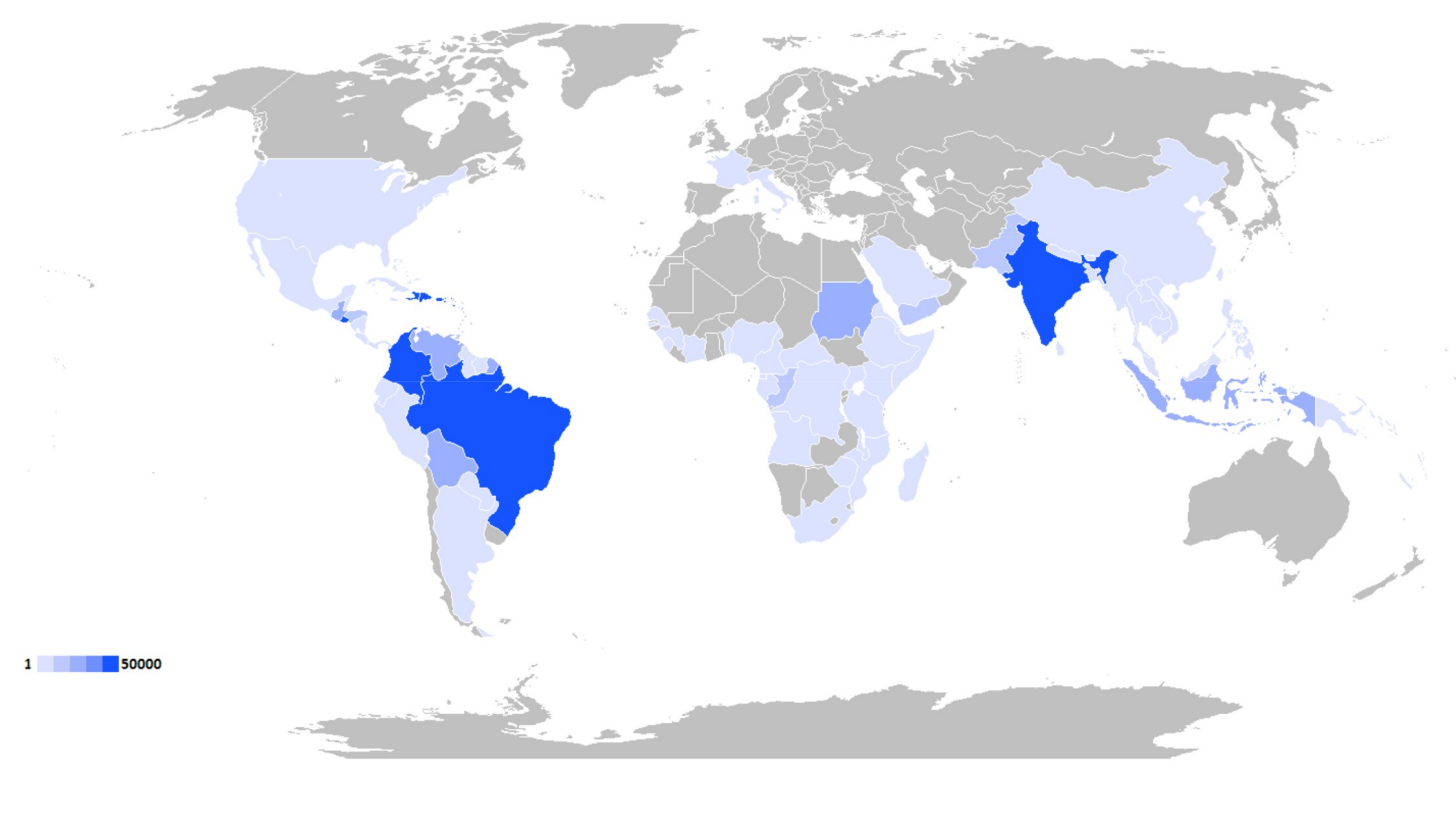
Global distribution of CHIKV. Countries having transmission of chikungunya, with gradient indicating average number of reported cases per year from 2010 to 2019. Map created using an open-access world map from Wikimedia Commons (https://commons.wikimedia.org/wiki/File:BlankMap-World.svg). CHIKV, chikungunya virus.

**Fig 3:**
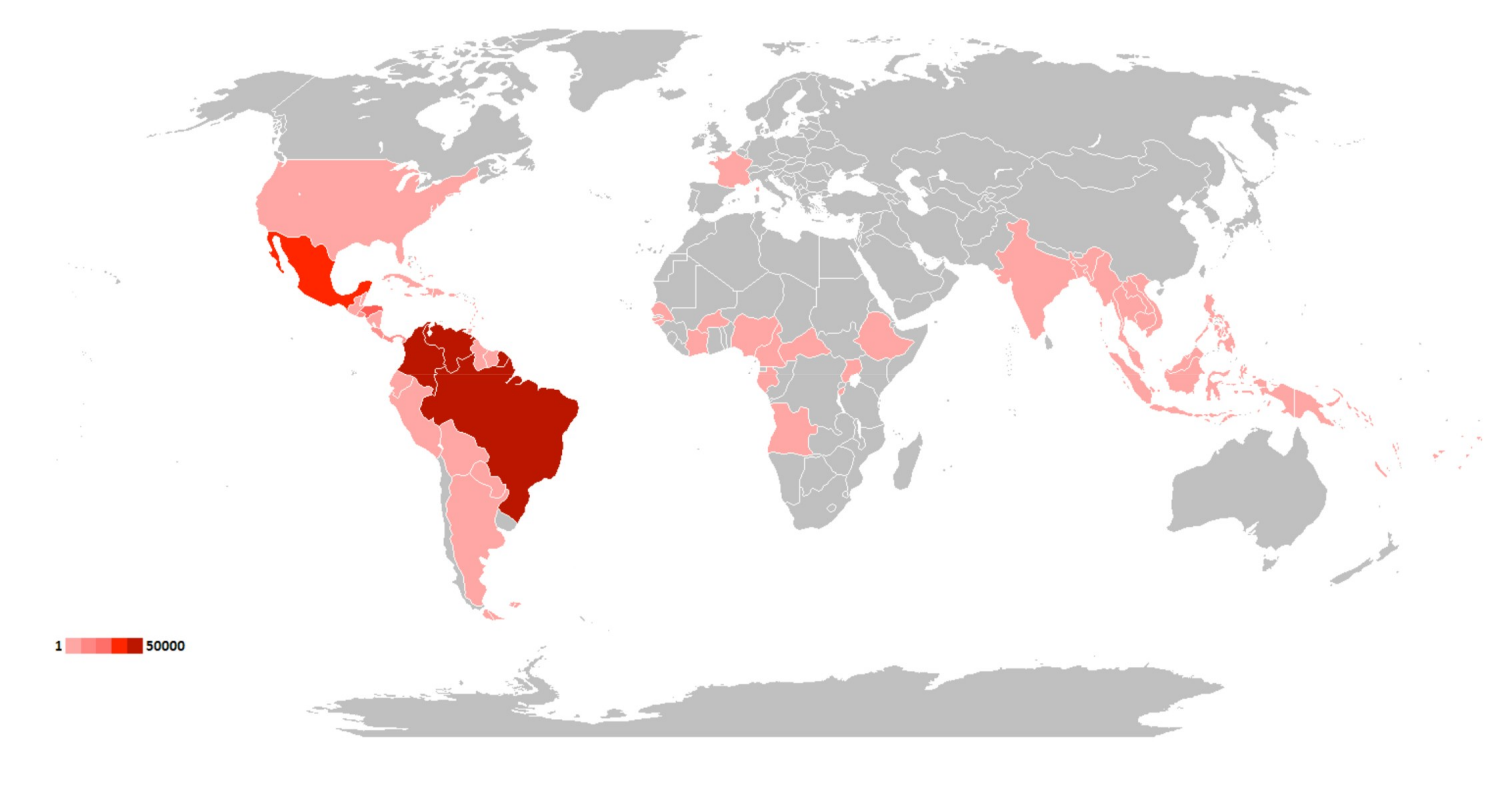
Global distribution of ZIKV. Countries having transmission of Zika, with gradient indicating average number of reported cases per year from 2010 to 2019. Map created using an open-access world map from Wikimedia Commons (https://commons.wikimedia.org/wiki/File:BlankMap-World.svg). ZIKV, Zika virus.

Although their related burden is unevenly distributed, both of the studied arboviruses affect populations across much of the globe. CHIKV was found to have spread more widely than ZIKV, with autochthonous transmission occurring in 114 countries and independent territories [8]. ZIKV was significantly less prevalent throughout the last decade, but reported autochthonous cases still occurred in 86 countries and territories [10]. As a result, over three quarters of the world’s populations now live in countries reporting endemic spread of CHIKV, and about half live in those reporting transmission of ZIKV. Figs 2 and 3 and Table 5 summarize the global distribution of the 2 viruses and the populations at risk for their continued spread.

**Table 5:** Populations of areas with endemic CHIKV and ZIKV transmission by WHO region.

Arbovirus	Number of affected territories and countries	WHO regions (populations of affected countries in thousands)	Total populations of affected countries (% global population)
CHIKV	114	**AFRO** (828,100) **AMRO** (929,765) **EMRO** (257,300) **EURO** (126,800) **SEARO** (1,898,600) **WPRO** (1,658,460)	5,699,025,000 (77.9%)
ZIKV	86	**AFRO** (445,600) **AMRO** (929,802) **EMRO** (0) **EURO** (64,300) **SEARO** (1,847,700) **WPRO** (263,880)	3,551,282,000 (48.6%)

AFRO, African region; AMRO, Americas region; CHIKV, chikungunya virus; EMRO, Eastern Mediterranean region; EURO, European region; SEARO, Southeast Asia region; WPRO, Western Pacific region; ZIKV, Zika virus.

The populations listed were quantified according to the 2015 estimates given in the United States Census Bureau’s International Database (https://www.census.gov/data-tools/demo/idb/).

## Discussion

Arboviral infections are known to cause a wide spectrum of acute disease, chronic complication, and mortality in many tropical and subtropical locales [16]. Among these diseases, chikungunya and Zika have been particularly notable for their rapid emergence and spread throughout several regions over the last decade, including the densely populated Americas [5–10]. Having infected millions of people since 2010, the 2 viruses have created a considerable disease burden, yet this burden has been largely understudied, and as a consequence, it has been omitted from most reports of international disability estimates [39,41,44,65]. Despite early evidence from 2014 suggesting that CHIKV could cause more burden than any other arbovirus in the Americas, quantified DALY estimates have been strikingly absent from the literature [71]. The most recent GBD report published by the IHME includes data for only 3 arboviral diseases: dengue, yellow fever, and Zika [39]. This report excludes chikungunya and exclusively assesses Zika-related burden in 2019, missing the years during which the most significant outbreaks occurred globally. To address this gap in awareness, we now report what are, to our knowledge, the first multiyear global DALY estimates for ZIKV and the first updated estimates for CHIKV in the last decade. Our systematic review and subsequent calculations of CHIKV- and ZIKV-related burden indicate that the 2 arboviruses cause significant health burdens at both regional and global levels. The burden caused by these viruses should be included in routine reports and regularly acknowledged in discussions of policy and containment priorities.

Our DALY estimates for CHIKV align with those reported by Cardona-Ospina and colleagues, who analyzed chronic CHIKV-linked burden in Latin America in 2014 and noted that DALYs attributable to CHIKV likely outweighed those of any other arbovirus in the region that year [71]. In our current analysis, we estimated that the virus has caused the annualized loss of over 158,000 DALYs in AMRO since its emergence. This value confirms that CHIKV is among the most problematic arboviruses in the region, causing a burden second only to that of dengue virus (DENV), which WHO estimated to cause the loss of 203,000 DALYs in its 2016 GBD report [38]. We estimate that ZIKV has caused an annualized DALY burden of 85,000 in AMRO since its emergence there in 2015. This estimate aligns with those previously published for Latin America and far exceeds the 23,000 DALYs associated with yellow fever in AMRO [38,72]. Although our CHIKV and ZIKV burden estimates for other regions are smaller in comparison, the differences may be partially explained by irregular surveillance and reporting in other regions. Since our calculations are based on nationally or regionally reported case counts, burden is inevitably underestimated in areas with limited surveillance, as is the case in many countries with continuing endemic and epidemic transmission outside the Americas [40,42,73,74]. Despite likely underestimation, our annualized value of 44,310 DALYs for global ZIKV burden still far exceeds the 2019 DALY estimate published by WHO and IHME of 347 DALYs [35]. This large discrepancy results mostly from the time periods analyzed. ZIKV infection rates were substantially higher from 2015 to 2018 than in 2019. These years of peak infection rates are not reflected in the estimate published by WHO and IHME. Further, a large portion of the ZIKV-associated burden we report is linked to the psychological symptoms experienced by mothers and caretakers of children affected by CZS, which is omitted from the 2019 GBD estimate.

The wide ranges of our estimated burden values, presented in Tables 4 and 5, reflect the current uncertainty in predicting the short- and long-term outcomes of infections, inconsistencies in published findings, and inherent challenges in generating yearly DALY estimates for arboviral diseases. DALY calculations require the use of annualized mortality rates and incidence rates of both acute and chronic manifestations of disease. Like most tropical diseases, CHIKV and ZIKV disproportionately affect resource-poor areas where healthcare and public health surveillance are often limited [2,15,40,41]. Due to inconsistencies in diagnostics and likely underreporting in endemic areas, it is impossible at this time to know precisely the number of symptomatic infections globally or their associated cause-specific deaths. Likewise, due to limited long-term data, it is currently unclear how many chronic cases either virus has caused, or the duration for which patients generally experience long-term complications. In particular, more follow-up will be needed to ascertain the lifetime prognosis of CZS, a condition which has only been studied since its discovery in 2015 [23,26]. Although it does so less frequently than ZIKV, CHIKV can also cause life-threatening complications in neonates via intrapartum infection and subsequent encephalitis. Such findings were well documented during the Réunion Island outbreak in 2005 and 2006, yet little information on nonfatal outcomes from neonatal CHIKV has been reported during outbreaks in the last decade [19–21]. As a result, any chronic sequelae due to neonatal infection represent sources of burden absent from our CHIKV DALY estimates.

The transmission patterns of arboviral infections further complicate the determination of their associated burden. Arboviruses are typically spread in epidemics, and information on interepidemic transmission is often unavailable. This tendency can, in part, explain the lack of sufficient data from regions outside AMRO that detail CHIKV and ZIKV incidence during the past decade. As evidenced by seroprevalence and case study data, several countries in Africa, Asia, and the Pacific had autochthonous spread of the 2 viruses between 2010 and 2019 [75–80]. For many of these nations, however, nearly no routine surveillance data are available. Given the findings of various seroprevalence studies and detected outbreaks in countries like Sudan, Yemen, India, and Thailand, it is very likely that a large number of infections occur across the world each year that are missed in global burden assessments [14,44,48,75,80,81]. Unequal distribution of disease burden is a potential limitation of a DALY-based approach, which requires a standardized methodology across regions. For example, the 92-year standard life expectancy used in the current GBD framework may not accurately reflect the life expectancy of individuals in the tropical areas affected most heavily by arboviral disease.

Even within the Americas, reporting guidelines differ greatly between countries. This inconsistency is perhaps most prominent in CZS diagnosis, for which surveillance in clinical settings can vary widely. By the end of 2017, over 85% of all CZS cases in the Americas were reported in Brazil, Guatemala, and the Dominican Republic, yet only about 47% of all ZIKV infections in the Americas were reported in these 3 nations [65]. This discrepancy between statistics indicates that many CZS cases were likely missed in areas that did not implement such proactive screening protocols. As illustrated in Table 5, even the relatively few known CZS cases contribute substantially more to overall global burden than all reported adult infections. Importantly, CZS-related burden is carried not only by affected children, but also by mothers and caretakers. The well-documented psychological symptoms experienced by mothers and caretakers are a unique effect of CZS, and the severity and frequency of these sequelae mean they must be considered when approximating the total burden caused by ZIKV [32,33]. Although nearly all diagnosed CZS cases have been in the Americas, confirmed cases have been reported in continental Africa and the Pacific [82,83]. Such findings suggest that CZS is in fact a global problem for which public health education and surveillance must be improved.

DALY estimates can provide a quantitative means by which to assess burden over a chosen period, but they fail to convey perhaps the most important characteristic of arboviral infections: outbreak potential. As a tool used by policymakers to assess burden during times of both average and peak demand, DALYs are traditionally calculated over a multiyear period. To derive estimates that can be readily compared to those available for other diseases, we analyzed CHIKV- and ZIKV-associated burden over the course of a decade rather than on a year-by-year basis. When averaged over the course of years, the burden associated with CHIKV, ZIKV, and other arboviruses can seem small relative to those caused by other pathogens. What is not conveyed by DALY estimates, however, is the often explosive spread of these diseases in epidemics that can overwhelm health systems and devastate local communities through supply chain dysfunction, economic downturn, and political disruption. As the rapid spread of CHIKV and ZIKV throughout the Americas illustrates, the introduction of arboviruses into new populations can result in spikes in cases that exceed the capacity of local health systems [84]. These surges in utilization during epidemics can indirectly result in additional burden, as patients with other medical conditions have reduced access to optimal treatment while health systems are overwhelmed.

The majority of the world’s populations now live in areas with evidence of arboviral transmission [8,10]. With climatic and social changes further driving the spread of *Aedes* mosquitoes to new regions, it is exceedingly likely that CHIKV, ZIKV, and other arboviruses will continue to cause explosive outbreaks in the near future [85,86]. Consequently, improved surveillance and preventative measures are important. Better estimates of the global burden and economic losses caused by arboviral epidemics point to the potential long-term cost-effectiveness of transmission-blocking strategies, including vector control and novel vaccine implementation.

## Supporting information

S1 PRISMA Checklist 2009 PRISMA checklist for systematic reviews and meta-analyses. PRISMA, Preferred Reporting Items for Systematic Reviews and Meta-Analyses.
(DOC)

S1 Table Listing of all included research articles. (XLSX)

S2 Table Listing of all included surveillance reports. (XLSX)

S3 Table Multi-way table showing CHIKV DALY range calculations. CHIKV, chikungunya virus; DALY, disability-adjusted life year.
(XLSX)

S4 Table Multi-way table showing CHIKV DALY discrete calculations. CHIKV, chikungunya virus; DALY, disability-adjusted life year.
(XLSX)

S5 Table Multi-way table showing ZIKV DALY range calculations. DALY, disability-adjusted life year; ZIKV, Zika virus.
(XLSX)

S6 Table Multi-way table showing ZIKV DALY discrete calculations. DALY, disability-adjusted life year; ZIKV, Zika virus.
(XLSX)

S1 Text Search algorithms used in systematic review. (DOCX)
