# Supplementary material for: Measuring the global burden of chikungunya and Zika viruses: A systematic review
Source: PLoS Negl Trop Dis. 2021 Mar 4;15(3):e0009055. doi: 10.1371/journal.pntd.0009055 (PMC7932082; doi:10.1371/journal.pntd.0009055)
Supplement: S1 Text — (DOCX) [file pntd.0009055.s008.docx]

**PubMed**:

(((chikungunya) OR (chikv) OR (zika) OR (zikv)) AND ((morbidity) OR (mortality) OR (incidence) OR (prevalence) OR (epidemiology) OR (outbreak) OR (complications) OR (disability) OR (quality of life) OR (DALY) OR (QALY)) ) AND (("2011"[Date - Publication] : "3000"[Date - Publication]))

**Web of Science:**

|  | (((ALL=chikungunya)  OR  (ALL=chikv)  OR  (ALL=zika)  OR  (ALL=zikv)  )  AND  ((ALL=morbidity)  OR  (ALL=mortality)  OR  (ALL=incidence)  OR  (ALL=prevalence)  OR  (ALL=epidemiology)  OR  (ALL=outbreak)  OR  (ALL=complications)  OR  (ALL=disability)  OR  (ALL=quality of life)  OR  (ALL=DALY)  OR  (ALL=QALY)  ))  Indexes=SCI-EXPANDED, SSCI, A&HCI, CPCI-S, CPCI-SSH, BKCI-S, BKCI-SSH, ESCI, IC Timespan=All years |
| --- | --- |

**Google scholar**:

"Chikungunya" OR "CHIKV" OR "Zika" OR "ZIKV" AND "morbidity" OR OR OR "mortality" OR OR OR "incidence" OR OR OR "prevalence" OR OR OR "epidemiology" OR OR OR "outbreak" OR OR OR "complications" OR OR OR "disability" OR OR OR "quality of life" OR OR OR "DALY” OR OR OR “QALY”

**LILACS**

CHIKV [Palavras] or ZIKV [Palavras] and morbidity [Palavras] or mortality [Palavras] or incidence [Palavras] or prevalence [Palavras] or epidemiology [Palavras] or outbreak [Palavras] or complications [Palavras] or disability [Palavras] or quality of life [Palavras] or DALY [Palavras] or QALY [Palavras] and 2010 [País, ano de publicação] or 2011 [País, ano de publicação] or 2012 [País, ano de publicação] or 2013 [País, ano de publicação] or 2014 [País, ano de publicação] or 2015 [País, ano de publicação] or 2016 [País, ano de publicação] or 2017 [País, ano de publicação] or 2018 [País, ano de publicação] or 2019 [País, ano de publicação] or 2020 [País, ano de publicação]

**African Journals Online**

"Chikungunya" OR "CHIKV" OR "Zika" OR "ZIKV" AND "morbidity” OR "mortality" OR "incidence" OR "prevalence" OR "epidemiology" OR "outbreak" OR "complications" OR "disability" OR "quality of life" OR "DALY” OR “QALY”

**SCIELO**

chikungunya [All indexes] or zika [All indexes] and morbidity [All indexes] or mortality [All indexes] or incidence [All indexes] or prevalence [All indexes] or epidemiology [All indexes] or outbreak [All indexes] or complications [All indexes] or disability [All indexes] or quality of life [All indexes] or DALY [All indexes] or QALY and 2010 [Publication year] or 2011 [Publication year or 2012 [Publication year] or 2013 [Publication year] or 2014 [Publication year] or 2015 [Publication year] or 2016 [Publication year] or 2017 [Publication year] or 2018 [Publication year] or 2019 [Publication year] or 2020 [Publication year]
